# Integrating Bio-Sensing Array with Blood Plasma Separation on a Centrifugal Platform

**DOI:** 10.3390/s23031710

**Published:** 2023-02-03

**Authors:** Snehan Peshin, Marc Madou, Lawrence Kulinsky

**Affiliations:** 1 Department of Mechanical and Aerospace Engineering, University of California, Irvine, CA 92697, USA; 2 School of Engineering and Science, Tecnológico de Monterrey, Monterrey 64849, Mexico

**Keywords:** centrifugal microfluidics, microfluidic valving, point-of-care diagnostics, Lab-on-CD

## Abstract

Numerous immunoassays have been successfully integrated on disc-based centrifugal platforms (CDs) over the last 20 years. These CD devices can be used as portable point-of-care (POC) platforms with sample-to-answer capabilities where bodily fluids such as whole blood can be used as samples directly without pre-processing. In order to use whole blood as a sample on CDs, centrifugation is used to separate red blood cells from plasma on CDs. There are several techniques for using specific fluidic patterns in the centrifugal fluidic network, such as reciprocation, that enhances the sensitivity of the immunoassays, including those using microarray antigen membranes. Present work demonstrates, for the first time, simultaneous integration of blood plasma separation (BPS) and reciprocation on the CD platform. The integrated design allows plasma that is separated from the red blood cells in a sedimentation chamber to flow into the reciprocation chamber via a narrow connecting channel of 0.5 mm × 0.5 mm cross-section. Due to the small cross-section of the connecting channel, there is no inflow of the red blood cell into the reciprocation chamber during subsequent fluidic operations of the CD. While no inflow of the red blood cells into the reciprocation chamber was observed, the conditions of 20 g jerk acceleration were also simulated in ANSYS finite element analysis software, and it was found that the CD design that was used is capable of retaining red blood cells in the sedimentation chamber. Experimentally, the isolation of red blood cells in the sedimentation chamber was confirmed using the ImageJ image processor to detect the visible color-based separation of the plasma from the blood. A fluorescent analyte testing on the bio-sensing array of the presented novel integrated design and on the standard reciprocation design CD was conducted for 7 min of reciprocation in each case. The test analyte was Europium Streptavidin Polystyrene analyte (10^−3^ mg/mL) and the microarray consisted of Biotin bovine serum albumin (BSA) dots. The fluorescent signals for the standard and integrated designs were nearly identical (within the margin of error) for the first several minutes of reciprocation, but the fluorescent signal for the integrated design was significantly higher when the reciprocation time was increased to 7 min.

## 1. Introduction

Centrifugal microfluidics in the last 20 years has developed to be one of the most promising platforms for inexpensive point-of-care (POC) diagnostics. It combines the advantages of portability, ease of automation, low cost, and facility of executing pre-processing of the samples with the capability of multiplexing multiple samples and/or multiple tests on the same disposable disc. Several process-specific centrifugal platforms currently exist on the market and perform various functions. Foremost among them are blood plasma separation (BPS) [1] and fast assaying through the reciprocation of fluid over sensor arrays [2]. Centrifugal force on the spinning disc facilitates fast density-based separation of the red blood cells from the plasma of the whole blood sample [2,3,4,5,6,7]. Centrifugal microfluidic discs (CDs) can also enhance the limit of detection in sensor array reactions by reciprocating fluid in the reaction chamber. This was achieved in the design that connected a fluid-reciprocating chamber with an air compression chamber [2]. In that design, the centrifugal force pushes the fluid outward into the air compression chamber at a higher angular velocity of the disc and when the disc rotation is slowed, the air compressed in the air compression pushes fluid back and that fluid goes back into the reciprocating chamber and washes over the microarray. By increasing and then decreasing the angular velocity of the disc, the plasma of the sample can be washed over the microarray multiple times, increasing the sensitivity of the assay. The plasma needed for the reciprocation step requires serial use of the BPS and reciprocation, which necessitates the use of separate CDs to perform each process and increases the cost of equipment and labor. Several papers attest to the efficacy of reciprocation and its specific implementations. The utilization of the air chamber to cause reciprocation by compression/decompression of the trapped air was first demonstrated by Noroozi et al. [8] A publication by Pishbin et al. [9] discusses the effects of moment of inertia and how reciprocation can enhance sensitivity of assays on CD. A more recent review of pneumatic operations on microfluidic centrifugal platforms [10] discusses the reciprocation and compares this fluid flow sequence to other fluidic methodologies. Finally, the latest paper by Hwu et al. [11] describes the implementation of reciprocation on a centrifugal microfluidic point-of-care diagnostic platform that employs a biosensing COVID antigen array.

In this paper, for the first time, a new CD design is demonstrated where blood plasma separation and reciprocation take place within the same sensor array chamber. The integration of these two processes eliminates the need for the transfer of plasma and speeds up the overall assay process. 

**Figure 1:**
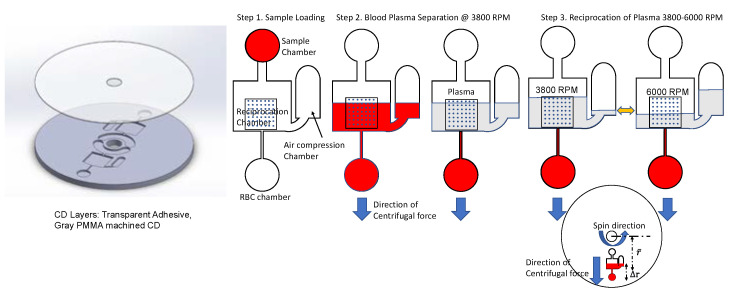
Design and operation of the integrated CD (inset on the bottom demonstrate the orientation of the fluidic network on the CD). Sequence of process steps include: Step 1—loading of the whole blood into the sample chamber; Step 2—spin at 3800 rpm for 4 min to separate red blood cells into the RBC chamber from the plasma in the reciprocation chamber and air compression chamber; Step 3—spin at 6000 rpm to compress the air in the air compression chamber and then the reduction of the angular velocity to 3800 rpm to push part of the plasma to the reciprocation chamber; repeated switching of the disc speed between 6000 rpm and 3800 rpm. Red color in the illustrations indicates the presence of red blood cells, while the gray color indicates plasma free from red blood cells.

**Figure 2:**
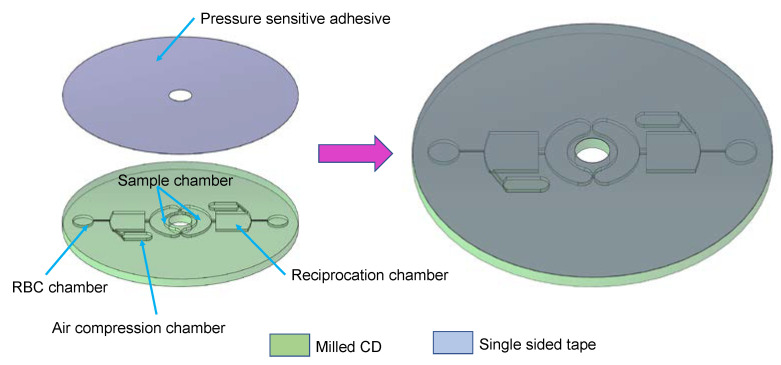
Fabrication and assembly of the integrated CD. The two layers—a milled polyacrylic CD (in green) and a single-sided tape (in gray)—are pressed together to fabricate the integrated disc. The polyacrylic CD (green) is milled to create the sample chamber, the RBC chamber, the reciprocation chamber, and the air compression chamber. The adhesive is punctured using pointed tweezers to create the vent holes and sample inlet holes.

**Figure 3:**
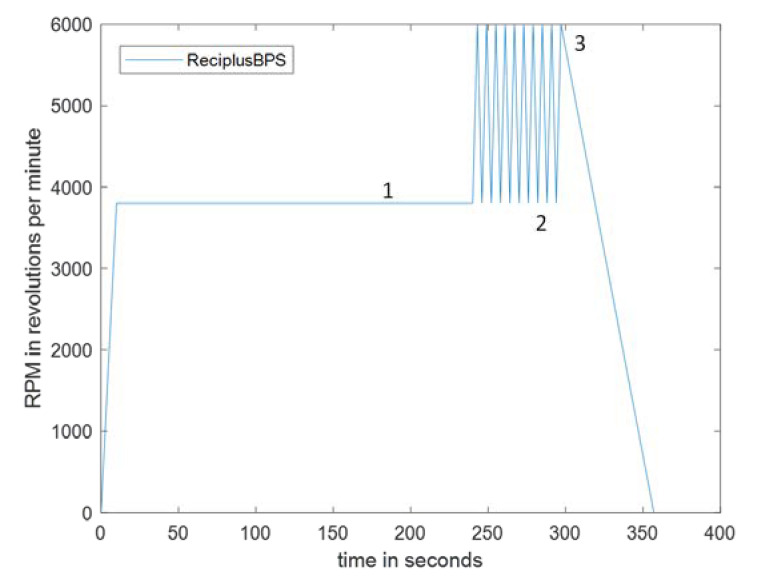
The spin profile of the integrated CD, which consists of (1) the plasma separation and red blood cells sedimentation, (2) the reciprocation regime implemented to pass the sample multiple times over the microarray in the reciprocation chamber, and (3) the slowing down of the spinning CD at 60 RPM/s.

In the integrated CD design (Figure 1 and Figure 2), the whole blood is loaded into a sample chamber. When the disc starts spinning, the blood sample is transferred to the Reciprocation Chamber. The reciprocation chamber is connected by a narrow channel to the RBC (red blood cell) chamber. The RBC chamber is designed to be fully filled with separated red blood cells (volume calculated using hematocrit ratio of blood). The volume of the RBC chamber is designed to be large enough to contain all the red blood cells from the blood sample after red blood cell separation even for patients with a high hematocrit ratio. Thus, the overspilling of the red blood cells into the reciprocation chamber is prevented. After the whole blood is pipetted into the sample chamber the disc is spun at 3800 revolutions per minute (rpm) for 4 min. During this step, the red blood cells sediment in the RBC chamber while the supernatant plasma collects in the reciprocation chamber. Thereafter, the disc is spun at 6000 rpm compressing the trapped air in the air compression chamber. Then the disc rotation is slowed to 3800 rpm again, enacting the reciprocation step when the plasma moves from the air compression chamber back into the reciprocation chamber. The repeated switching of the angular velocity of the disc between 3800 rpm and 6000 rpm (spin profile is presented in Figure 3) moves the plasma in and out of the reciprocation chamber where the microarray is located. 

Table 1 summarizes the comparison of the integrated plasma separation CD with the standard reciprocation protocol that uses a separate CD for blood plasma separation.

A purity comparison graph between commercial plasma preparation tubes and the large-volume centrifugal microfluidic device is given in an earlier publication by our group [1]. For the same duration of sedimentation time, the compact disk-based device yielded separated plasma of equal or greater purity than the tube format. For example, 2.5 min of CD processing yielded 99.9999% pure plasma compared to 58% when the tube format was used.

## 2. Materials and Methods

The CD was fabricated by milling a 3 mm thick polyacrylic sheet (8589K43 Clear Scratch- and UV-Resistant Acrylic Sheet, McMaster-Carr, Elmhurst, IL, USA) using a Tormach PCNC CNC milling machine (Tormach, Waunakee, WI, USA). SolidworksTM (Dassault Systèmes, Vélizy-Villacoublay, France) was used to design the fluidic network on the CD consisting of chambers and channels presented in Figure 2. The fluidic network consists of the sample chamber, the reciprocation chamber, the RBC chamber, and the air compression chamber. The reciprocation chamber is connected to the RBC chamber by a narrow channel with a cross-section of 500 microns by 500 microns. SolidCAM (SolidCAM, Newtown, PA, USA) was utilized to create G-code as an input for the CNC machine. The geometry of the integrated disc and its fluidic network is provided in Figure S1. After milling the CD, the pressure-sensitive tape (9795R tape, 3MTM, Saint Paul, MN, USA) is cut using the cutter plotter (Silhouette Cameo 4, Lindon, UT, USA) and then applied as a top layer of the CD. The vents and sample inlet apertures are punctured in the top layer after the disc is assembled. A defibrinated bovine blood (Quad Five, Golden Valley, MT, USA) is used for each test.

Here, 70 µL of bovine blood (no blood coagulation was observed within 10 min of our experiments) is pipetted into the sample chamber. The CD is run at 3800 RPM for 4 min for the red blood cells to sediment into the RBC chamber. The reciprocation sequence is then implemented where the spin rate is alternated between 6000 rpm and 6000 rpm (see the spin profile in Figure 3) as plasma moves between the air compression chamber and reciprocation chamber repeatedly washing over the microarray. Finally, the disc is slowly decelerated to a complete stop at a rate of 60 RPM/s to decrease the Euler force, which can potentially cause the transfer of some red blood cells to the reciprocating chamber. 

The testing performed for the proof-of-principle demonstration is that of the biotin-streptavidin conjugation. The Biotinylated Bovine Serum Albumin (2 mg/mL, Pierce™ Bovine Serum Albumin, Biotinylated, 29130, Thermofisher Scientific, Waltham, MA, USA) dots are printed on the nitrocellulose membrane (HF 120, 7 mm by 7 mm, Millipore Sigma, Burlington, MA, USA) in an array 7 by 7 of 100 μm dots applied using Biodot (BIODOT AD3050, Irvine, CA, USA) and that microarray is placed in the reciprocation chamber of the disc before its assembly. Phosphate Buffer Saline (PBS) (Thermofisher Scientific) solvent with 10^−3^ mg/mL dilution of Europium Streptavidin-coated Polystyrene microbeads (29470701011150, Fluoro-Max Fluorescent Streptavidin-Coated Particles, Thermofisher Scientific, Waltham, MA, USA) is loaded into the sample chamber. The reciprocation regime (see Figure 3) is run for three separate time intervals successively—for 2.5 min, 5 min, and 7 min. The tests were run on two separate discs: one with the standard reciprocation CD design (provided in Figure S2) and another disc is the integrated plasma separation and reciprocation CD. The original standard reciprocation setup was produced and tested in our lab. The standard reciprocation CD design was taken from the Noorozi paper [2] and we used the same materials and fabrication methods as those utilized for the integrated CD.

The efficiency of reciprocation for the integrated plasma separation design was compared to that of the standard design using (on each disc) the NC membranes spotted with biotinylated bovine serum albumin (BSA) in microarray format and dried in a silica gel chamber for one week before use. The sample loaded in the sample chambers contained 10^−3^ mg/mL of Europium-coated streptavidin polystyrene beads. The Europium beads were diluted in PBS and sonicated before use. 

**Figure 4:**
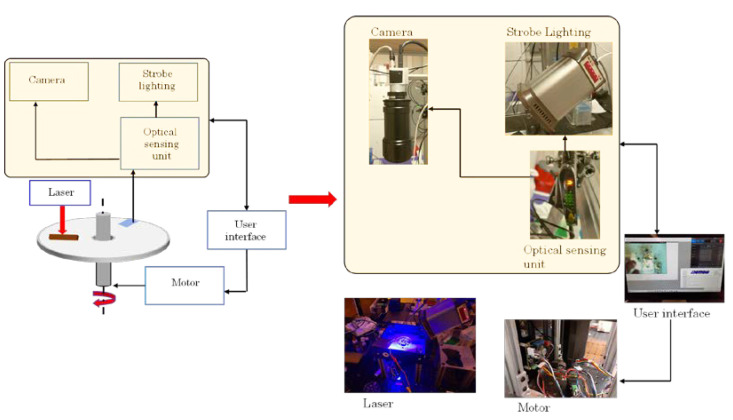
The experimental setup for testing the integrated CD consisting of the spin chuck that is spun by a motor and the motor is connected to the controller that gets its input for angular velocity and acceleration via graphic user interface on the computer. The optical sensing unit receives a laser light reflected from a piece of reflecting tape secured to the CD and this light serves as a signal to open the shutter to take a picture with digital camera and to shine the stroboscopic light to add illumination for taking the picture. This sequence of photographs (one per disc’s revolution) is recorded as a video that is captured from a computer screen using recording software Bandicam (Bandicam Company, Irvine, CA, USA). Reproduced from [12,15].

The spin stand setup for testing the integrated CD is presented in Figure 4. The integrated CD is placed on a custom-made spin chuck and secured with a nut in the center of the spinning axis. The disc is spun by a motor (SM3450 D, Motion USA, Columbus, OH, USA) and controlled by a motor controller (BLDC Servo Motor Controller, EZSV23/EZSV17, AllMotion, Union City, CA, USA) [12]. A stroboscopic light source (DT-311A, Shimpo, Lynbrook, NY, USA) is used to illuminate the spinning disc during picture taking with a digital camera (acA800-510uc, Basler AG, Ahrensburg, Germany). The light source shines down on CD and this light gets reflected from a piece of reflecting tape secured on the spinning disc. This reflected light is sensed by an optical sensor (D10DPFP, Banner Engineering Corp., Minneapolis, MN, USA) and is used for the synchronization of taking pictures (opening the shutter of the digital camera) and illumination by the stroboscopic light of the CD. Thus, one image is taken per one revolution of the CD. This sequence of photographs is captured as a video from a computer screen utilizing the video-capturing software Bandicam (Bandicam Company, Irvine, CA, USA). ImageJ (https://imagej.nih.gov/ij/index.html, National Institute of Health, Bethesda, MD, USA) is used for image processing to identify whole blood, red blood cells, and plasma that is free from red blood cells [13,14].

## 3. Theory

In centrifugal microfluidics, the fluidic propulsion is forced primarily by the centrifugal force:

(1) $$P_{cent}=\rho \Delta r\bar{r}\omega^{2}$$

where, $P_{cent}$ is the pressure on the fluid due to the application of centrifugal force (in Pa), ρ is the density of the fluid (in kg/m^3^), $\bar{r}$ is the average radial distance of the fluid column from the center of the CD (in m), ∆r is the difference between the distances from the disc’s center and the top of the fluid column and the corresponding distance to the bottom of the fluid column (in m), and ω is the angular velocity of the disc (in rad/s). The illustration of several distance terms used in Equation (1) is presented in the inset of Figure 1.

According to Equation (1), denser items in the suspension of various phases will be pushed toward the periphery of the disc with greater force than the lighter objects, causing the denser red blood cells to sediment in the RBC chamber, while lighter plasma rises up toward the top of RBC chamber and then is completely pushed up into the reciprocation chamber (see Figure 1). This density-based separation is caused by the buoyancy principle: if left in the stational beaker, the red blood cells will sediment to the bottom of the beaker with time. The speed of such sedimentation is greatly increased on the spinning disc where the gravitational acceleration can be greatly surpassed by the radial acceleration a:

(2) $$a=\omega^{2}r$$

where ω is the angular velocity of the disc and *r* is the distance to the center of the disc.

For example, if the disc is spinning at the angular velocity of 3800 rpm, at a distance of 2 cm away from the disc’s center, the radial acceleration is roughly equal to 323 g. Therefore, the red blood cell separation takes a few minutes only and the supernatant plasma free of red blood cells fills the reciprocation chamber where the microarray is located. The narrow channel connecting the RNC chamber and the reciprocation chamber prevents the inflow of red blood cells into the reciprocation chamber under any fluidic regimes tested in this work. Moreover, the simulation (see Section 5 below) also demonstrates that even at the more extreme jerk motion (abrupt changes between the spin directions), the fluidic design of the integrated disc will guarantee retention of the sedimented red blood cells in the RBC chamber.

## 4. Results and Discussion

### 4.1. Blood Plasma Separation

The integrated disc that was loaded with the whole bovine blood (see Section 2 above) was initially spun at 3800 rpm to perform sedimentation of the red blood cells and then 10 cycles of alterations between 3800 rpm and 6000 rpm were performed to compress and decompress the air in the air compression chamber that resulted in repeated plasma reciprocation over the biosensing array placed in the reciprocation chamber. The spin program (see Figure 3) concludes with a 60 s ramp-down of the spin speed at a rate of 60 rpm/s to a complete stop. 

**Figure 5:**
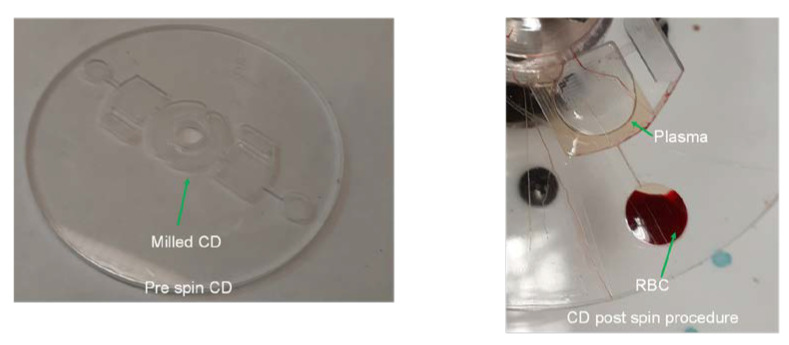
(**Left**) The image of the fabricated CD before the blood sample is loaded. (**Right**) The image of the part of the disc with RBC chamber and the reciprocation chamber after blood plasma separation has been performed—the red blood cells are all separated in the RBC chamber as is evidenced by the dark red color, while plasma only (transparent fluid) is present in the reciprocation chamber. The nitrocellulose membrane that is normally present in the reciprocation chamber has been removed to improve the clarity of the image.

**Figure 6:**
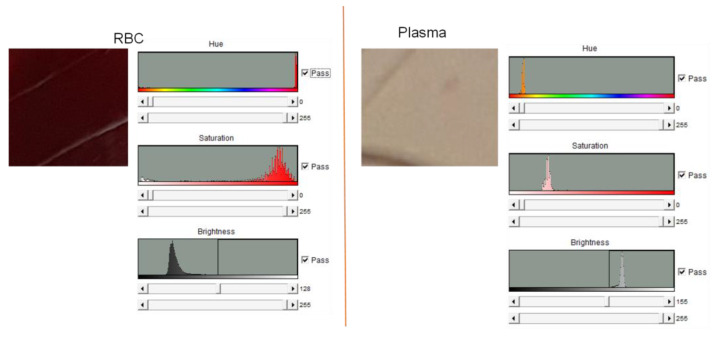
Image analysis performed with ImageJ after the completion of the spin program that separates the red blood cells. Images of the RBC chamber (on the **left**) demonstrate the strong presence of red hue of the red blood cells that are absent in the image analysis of the reciprocation chamber (on the **right**).

After performing this spin program, we observe the complete separation of red blood cells into the RBC chamber and of clear plasma into the reciprocation chamber as seen in Figure 5. The images taken by the digital camera were analyzed with ImageJ software to confirm the complete separation of the red blood cells from the plasma. A similar process has been used by Hwu in his measurements of biosensing arrays in a recent paper [11]. Figure 6 (left) demonstrates that the contents of the RBC chamber are saturated with a red hue, while the plasma in the reciprocation chamber (Figure 6, right) has the same hue as the spin chuck underneath (i.e., that the plasma is transparent and devoid of the red blood cells). 

Integrated plasma separation disc while effectively isolating the separated red blood cells from the reciprocation chamber, also utilizes a simpler disc design as compared to the standard reciprocation disc (see the disc designs presented in Figures S1 and S2).

### 4.2. Simulation

ANSYS Fluent (ANSYS, Canonsburg, PA, USA) simulation software was utilized to simulate the flow of blood and model the process of red blood cell separation where the RBC chamber (in red) is filled with red blood cells. The point of special emphasis is the narrow fluidic channel connecting the RBC chamber to the reciprocation chamber as we checked for the movement of the red blood cells out of this channel into the reciprocation chamber.

**Figure 7:**
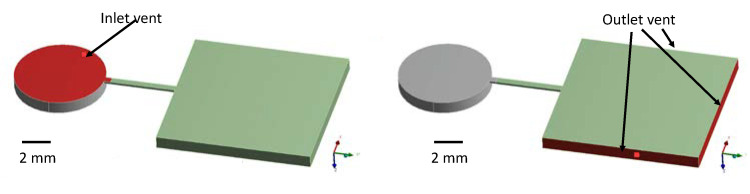
The Fluent FEA simulation was performed with the boundary conditions of the atmospheric pressure at the inlet vent for the whole blood sample (**left**) and the outlet vents along the side walls of the RBC chamber (**right**).

**Figure 8:**
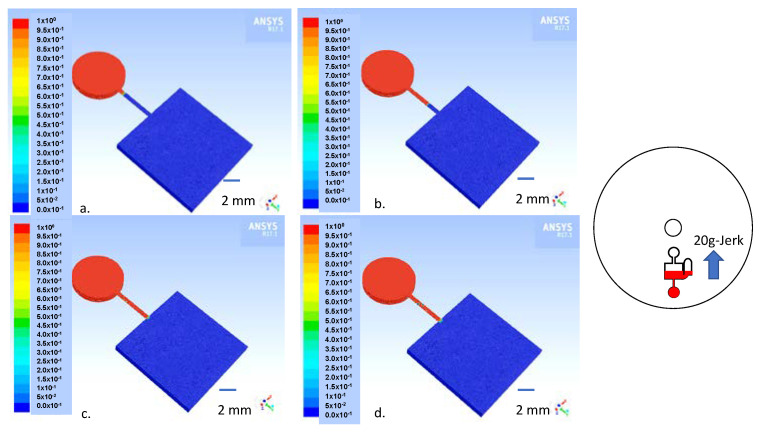
The results of two-phase flow Fluent simulation (going from stop frame (**a**–**d**)) that models the jerk acceleration of 20 g directed toward the disc’s center (see the inset illustration) where the red blood cells that are separated in the rectangular RBC chamber (red) do not enter into the reciprocation chamber that is filled with supernatant plasma (blue) as the red blood cells are pinned at the connecting channel—reciprocation chamber junction.

In order to test the robustness of the isolation of the separated red blood cells in the RBC chamber, we modeled the situation of sudden jerk acceleration of the disc in the radial direction, which causes red blood cell acceleration of 20 g toward the disc’s center. A transient laminar two-phase flow of air and red blood cells is modeled with properties of the whole blood summarized in Table 2. The finite element analysis (FEA) is carried out in time steps of 0.001 s. The inlets and outlets maintained at atmospheric pressure are presented in Figure 7. The volume fraction of the red blood cells of 1 is maintained at the inlet. The red color in Figure 8 represents the volume fraction of blood as 1 and air as 0, and the blue hue represents the volume fraction of blood as 0 and air as 1. The simulation results in Figure 8 demonstrate that the red blood cells that flow into the connecting channel are stopped at the interface between the reciprocation chamber and the connecting channel pinned by the capillary expansion valve due to the high surface tension of the whole blood. The simulation result suggests that the narrow connecting channel provides sufficient isolation for the separated red blood cells without the need for additional valving methods. Indeed, in our handling of the disc with the separated red blood cells, we were unable to dislodge the separated red blood cells from the RBC chamber. The details of the simulation framework are presented in our previous paper describing wax valving on centrifugal microfluidic platforms [12].

### 4.3. Efficiency of Reciprocation

**Figure 9:**
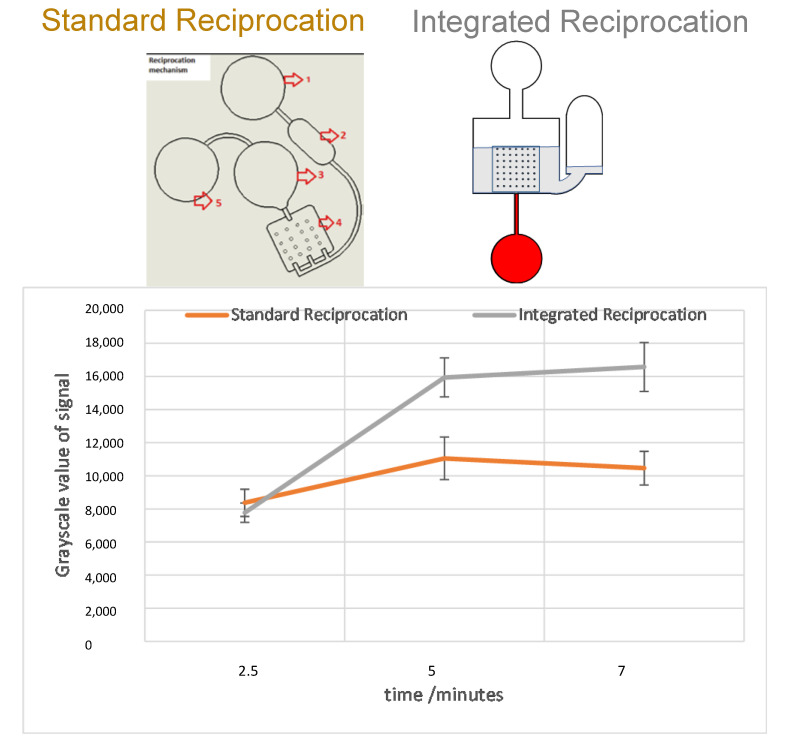
Comparison of the integrated reciprocation versus standard reciprocation with process times. We used Europium-coated streptavidin particles as the analyte to provide a signal when reacting with NC membrane-coated biotinylated BSA.

The reciprocation tests were performed with the suspension of Europium microbeads (see Section 2 above). The standard reciprocation disc [2] and the integrated plasma separation discs used the reciprocation regime (see Section 2) for 2.5 min, 5 min, and 7 min, and the images of microarrays were analyzed with ImageJ software to obtain the grayscale intensity of the images. The obtained results are plotted in the graph presented in Figure 9. For both, standard reciprocation and the integrated disc design, the maximum signal is reached approximately within 5 min of reciprocation. It is clear from the data that the sensitivity of the integrated design is not inferior to the standard reciprocation design. It can be seen that for the longer reciprocation time, the integrated design performs better than the standard design. The likely reason for the superiority of the integrated plasma separation design is that, in this design, the sample drains completely from part of the microarray, while in the standard reciprocation design, the microarray is completely covered with the sample during the reciprocation process. The partial draining and re-filling of the reciprocation chamber causes boundary layer removal and reformulation over the microarray during each reciprocation cycle and the stagnant layer over the microarray is avoided, increasing the overall sensitivity of the assay. 

Various angular velocity profiles, sample volumes, and reciprocation process times were tested in an approach modeled on that by Naroozi et al. [2,8,19] to settle on process parameters that yield optimal results. Optimization of the angular velocity of the disc to achieve blood plasma separation avoiding both, blood coagulation and lysis is demonstrated in Table 3.

The results presented in the current work would help in designing Lab-on-CD point-of-care diagnostic platforms that utilize blood samples as well as other liquid samples requiring density-based fractionation. Integration of sample preprocessing leads to less expensive and faster assays. Less expensive and faster point-of-care diagnostic platforms have gained new importance in an age of mass testing prompted by the COVID pandemic [11]. The demonstrated integrated sample preparation approach can be used in other applications, such as the detection of antibiotic resistance shown in previous work by Perebikovsky et al. [20].

## 5. Conclusions

A novel efficient CD efficient design is presented where two processes—(a) a centrifugal separation of the supernatant red-blood-cell-free plasma from the whole blood and (b) sample reciprocation that increases assay sensitivity are unified and integrated on a single disc. The complete separation of the red blood cells into the RBC chamber is demonstrated with image analysis with ImageJ software. The narrow 0.5 mm by 0.5 mm channel that connects the RBC reservoir with the reciprocation chamber (where the test microarray is located) is effective for blocking the red blood cells from entering the reciprocation chamber. Prevention of the accidental backflow of the red blood cells into the reciprocation chamber is not only evidenced experimentally, but the ANSYS simulation results of the jerk reverse acceleration of 20 g also exhibit complete retention of the red blood cells in the RBC chamber. The image analysis with ImageJ software was used to establish that the maximum signal is reached within 5 min of reciprocation. It was demonstrated that the sensitivity of the biotin-streptavidin conjugation-based test was the same for the standard reciprocation and for the integrated designs, while for 5 min and 7 min of reciprocation, the sensitivity of the integrated design test was superior to the test with the standard reciprocation design. Therefore, the integrated whole blood plasma separation CD design could be utilized for the implementation of more effective on-disc processes for point-of-care applications [21].

## Figures and Tables

**Table 1:** Comparison of Integrated blood plasma separation with Standard Reciprocation system that uses two CDs [1,2].

Property Type (Units)	Standard Reciprocation [1,2]	Integrated Reciprocation
Process time (minutes)	25	7
Sample Volume (mL)	1	0.02
Number of CDs required	two separate CDs	two processes on a single CD
Needs Trained Personnel	No	No

**Table 2:** Properties of the whole blood used in the FEA simulation describing the separation and isolation of the red blood cells in the RBC chamber.

Property Type (Units)	Value
Contact angle of blood (with acrylic) [degrees] [14] θ_c_	70
Density of blood [kg/m^3^] [16] ρ	994
Surface tension of blood [N/m] [17] γ	0.053
Viscosity of blood [N·s/m^2^] [18] µ	0.0045

**Table 3:** Optimization of the angular velocity of the disc (in revolutions per minute) for blood plasma separation process.

CD Sample Number	RPM	Process Time/Lysis
1	1500	10 min/Coagulation
2	2500	8 min
3	3800	6 min
4	5500	Lysis

## Data Availability

Not applicable.

## Supplementary Materials

The following supporting information can be downloaded at: https://www.mdpi.com/article/10.3390/s23031710/s1, The supplementary shows the dimensions of the various chambers on the CD in Figure S1. In Figure S2, it shows the design of the standard reciprocation CD from Noroozi et. al.
