# Supplementary figures and images for: Integrating Bio-Sensing Array with Blood Plasma Separation on a Centrifugal Platform
Source: Sensors (Basel). 2023 Feb 3;23(3):1710. doi: 10.3390/s23031710 (PMC9920851; doi:10.3390/s23031710)

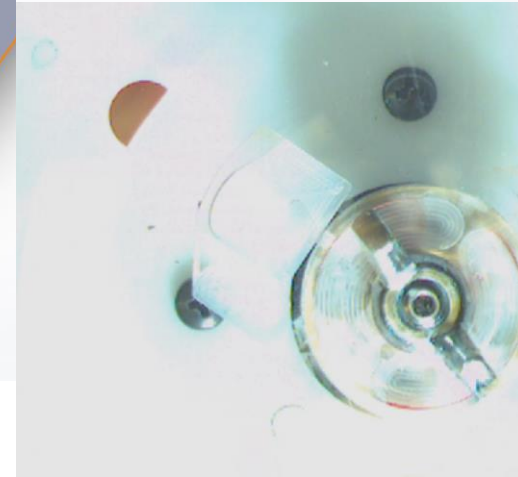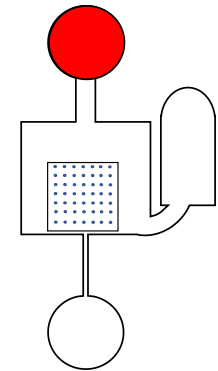

## APPENDIX S2, Dimensions of the standard CD on a 8 cm diameter PMMA CD, Noorozi et. al

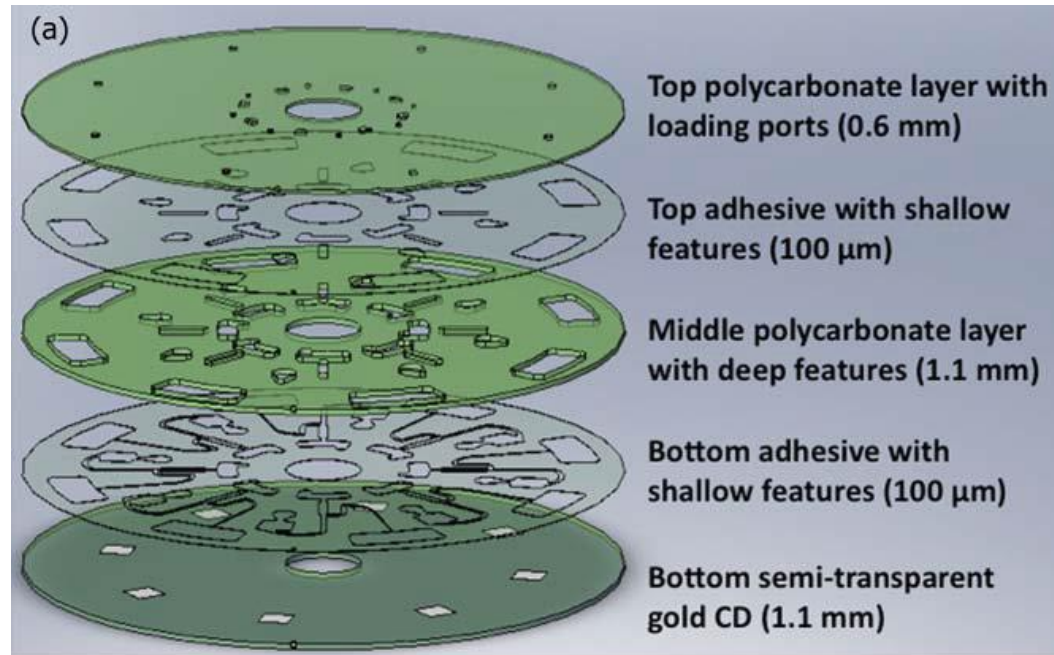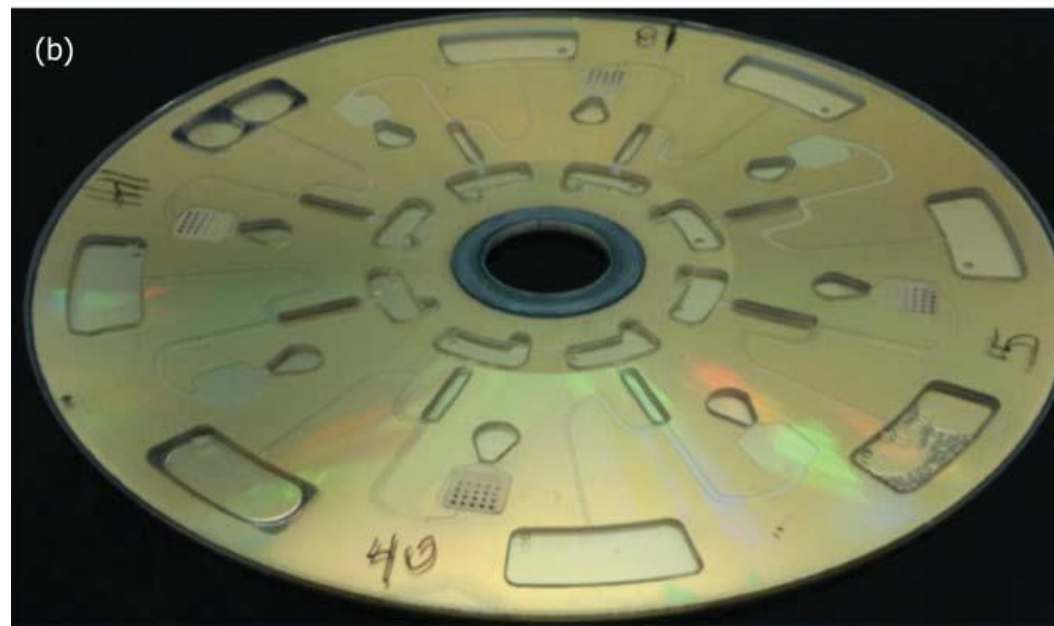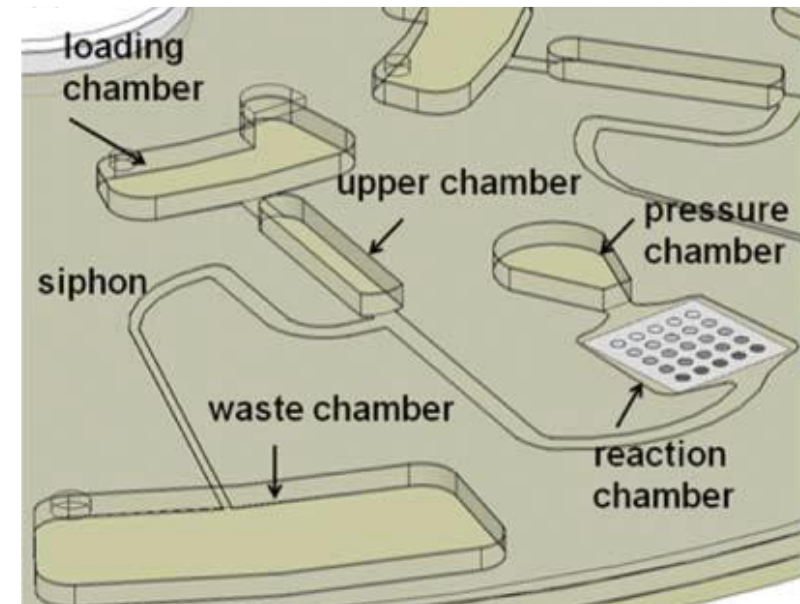

Supplement: Supplementary file 1 [file sensors-23-01710-s001.zip › sensors-2176511-supplementary.pdf]
